# Hepatic Subcapsular Hematoma: A Rare Complication After ERCP: A Case Report and Review of Literature

**DOI:** 10.1002/ccr3.71525

**Published:** 2025-11-25

**Authors:** Anwar Zahran, Amin Khader, Firas Besharieh, Saja Amer, Ahmed Nofal, Mohammed AbuBaha

**Affiliations:** ^1^ Department of Medicine An‐Najah National University Nablus Palestine; ^2^ Thabet Thabet Hospital Tulkarm Palestine; ^3^ Department of Surgery, General Surgery Resident, Thabet Thabet Hospital Tulkarm Palestine

**Keywords:** endoscopic retrograde cholangiopancreatography, ERCP complication, gallstone pancreatitis, infected hematoma, laparoscopic cholecystectomy, subcapsular hepatic hematoma

## Abstract

Endoscopic retrograde cholangiopancreatography (ERCP) is widely used for diagnosing and treating biliary and pancreatic disorders, yet it carries a risk of complications, including the rare development of subcapsular hepatic hematoma (SHH). We report the case of a 32‐year‐old woman who presented with abdominal pain following ERCP for gallstone pancreatitis. She was afebrile and hemodynamically stable but appeared pale and exhibited right upper quadrant and epigastric tenderness. Laboratory evaluation revealed a hemoglobin drop to 8.6 g/dL. Computed tomography demonstrated a large right‐lobe subcapsular hepatic hematoma measuring 15.5 × 7.5 cm with associated air locules. Despite initial conservative management, the hematoma enlarged, and the patient subsequently underwent laparoscopic cholecystectomy with evacuation of the infected hematoma 3 days after admission. This case highlights the importance of early clinical vigilance and prompt imaging in the detection of post‐ERCP complications. Although conservative therapy may be effective in stable cases, surgical intervention becomes essential when complications progress or infection develops.

## Key Clinical Message

A rare but possibly fatal side effect of ERCP is subcapsular hepatic hemorrhage. Early detection by imaging and careful observation is essential for prompt identification. Even though conservative treatment is often successful, in order to minimize serious morbidity and improve patient outcomes, prompt surgical intervention is necessary in situations of infection or progression.

**Abbreviations**
CBD: common bile duct
CT: computed tomography
ERCP: endoscopic retrograde cholangiopancreatography
RUQ: right upper quadrant
SHH: subcapsular hepatic hematoma

## 1 Introduction

**FIGURE 1:**
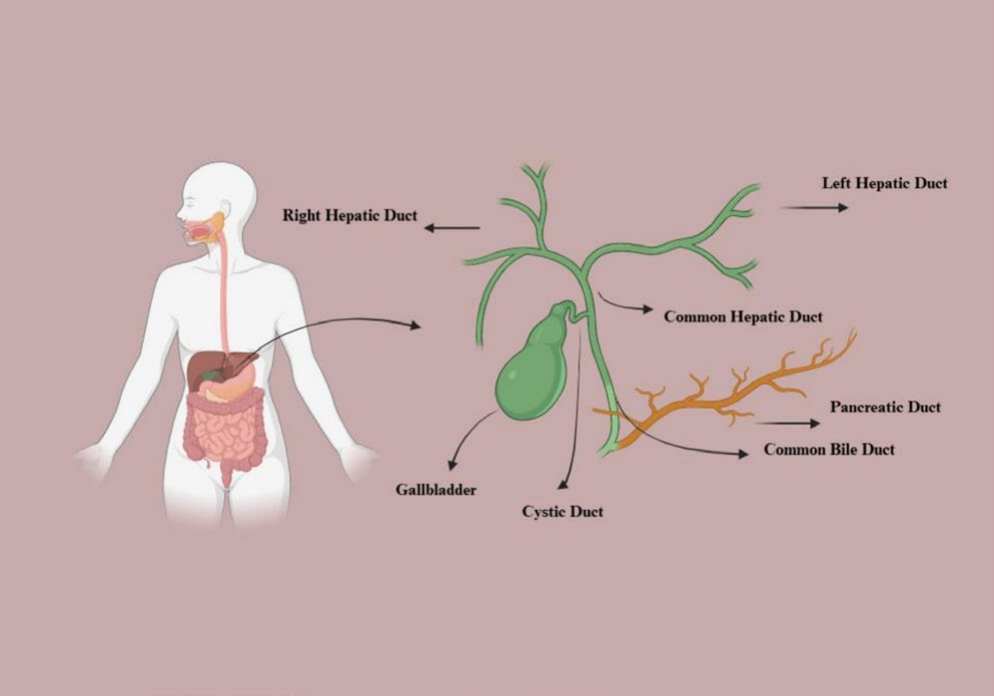
Schematic illustration of the biliary system anatomy, showing the right and left hepatic ducts joining to form the common hepatic duct, the cystic duct from the gallbladder, and their convergence into the common bile duct with connection to the pancreatic duct.

One of the most common minimally invasive techniques for diagnosing and treating biliary and pancreatic disorders, such as choledocholithiasis, gallstone pancreatitis, and biliary strictures, is endoscopic retrograde cholangiopancreatography (ERCP) [1] (Figure 1).

Even if performed by well‐experienced professionals, ERCP‐associated complications are around 2.5%–8%, with a 0.5%–1% mortality rate range [2]. Associated bleeding complications can range from mild traumatic mucosal to massive symptomatic blood loss requiring intensive care and multiple transfusions. Patients with coagulopathy and patients on anticoagulants or antiplatelet agents are at higher risk of bleeding complications [3].

Only 20 occurrences of subcapsular hepatic hematoma (SHH), a rare complication that may nonetheless be fatal after ERCP, have been reported [3]. Here, we report the case of a 32‐year‐old woman who had a subcapsular hepatic hematoma after presenting with stomach discomfort after ERCP. We also outline the case's medical and surgical treatment.

This work has been reported in line with the SCARE criteria [4].

## 2 Case Presentation

The thirty‐two‐year‐old white woman presented to the emergency department of a Palestinian hospital complaining of epigastric pain associated with nausea and vomiting. The patient has no significant medical history other than a previous history of biliary pancreatitis that was managed conservatively.

Upon admission, the patient was afebrile with normal vital signs. The patient showed an unremarkable physical examination except for forepigastric tenderness. Ultrasonographic findings included cholelithiasis, choledocholithiasis, mild dilatation of intrahepatic ducts, a common bile duct (CBD) measuring up to 1.4 cm, and an edematous pancreas surrounded by mild free fluid.

**FIGURE 2:**
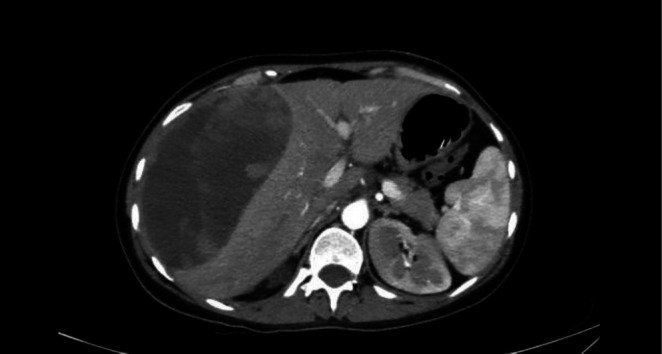
Common bile duct dilatation suggesting obstructive jaundice.

Her laboratory workup showed white blood cells 9 × 1.000/μL (4–10 × 1.000/μL) with 82% neutrophils, hemoglobin 12.4 g/dL (12.5–15.5 g/dL), platelets 331 × 1.000/μL (150–450 × 1.000/μL), bilirubin 2.2 mg/dL (0.3–1.2 mg/dL), with a direct bilirubin of 1.1 mg/dL (0–0.3 mg/dL), aspartate aminotransferase 122 U/L (2–40 U/L), alanine aminotransferase 79 U/L (4–49 U/L), alkaline phosphatase 260 μ/L (32–92 μ/L), lipase 8740, amylase 3124, prothrombin time ratio 16 (11–13.5), international normalized ratio 1.2 (0.8–1.2), and activated partial thromboplastin time ratio 34 (25–35). A computed tomography (CT) scan was ordered, and common bile duct dilatation was found (Figure 2).

For the next 3 days, the patient received conservative treatment consisting of 500 mg of metronidazole three times a day and 400 mg of ciprofloxacin twice a day. However, the patient was sent to another hospital for ERCP with sphincterotomy and CBD stone extraction without a stent, after which they were returned to our hospital.

**FIGURE 3:**
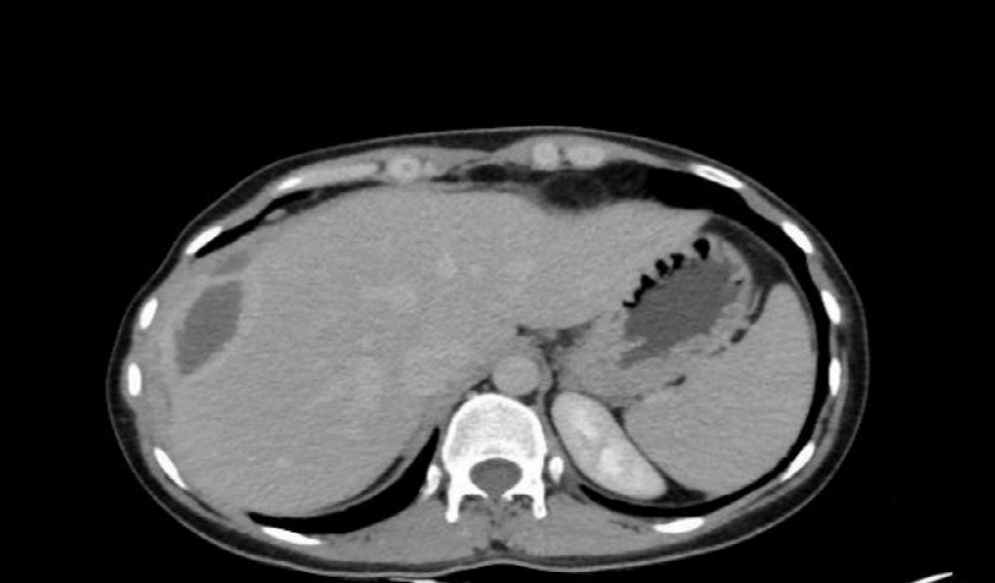
A right hepatic lobe SHH with air locules.

The patient began complaining of right upper quadrant (RUQ) discomfort and vomiting around 24 h following the ERCP. The patient appeared pale upon examination with RUQ and epigastric discomfort, was hemodynamically stable and afebrile, and her laboratory results revealed a decrease in hemoglobin to 8.6 g/dL. An ultrasound revealed a heterogeneous development in the RUQ measuring 14 by 7 cm. Consequently, a computed tomography (CT) scan was done, revealing a right hepatic lobe SHH measuring 15.5 × 7.5 cm with air locules generating a mass effect on the liver (Figure 3). With intravenous fluids, packed red blood cell transfusions, fresh frozen plasma, serial hemoglobin testing, and Meropenem 1 g three times a day in place of the prior antibiotic regimen, the patient was maintained under conservative care.

Serial abdominal ultrasounds in the following week showed no change in the size of the hematoma. After the regression of the patient's symptoms, and stable hemoglobin levels ranging from 8.6 to 9.1 g/dL, she was discharged with instructions to follow up at the outpatient clinic.

**FIGURE 4:**
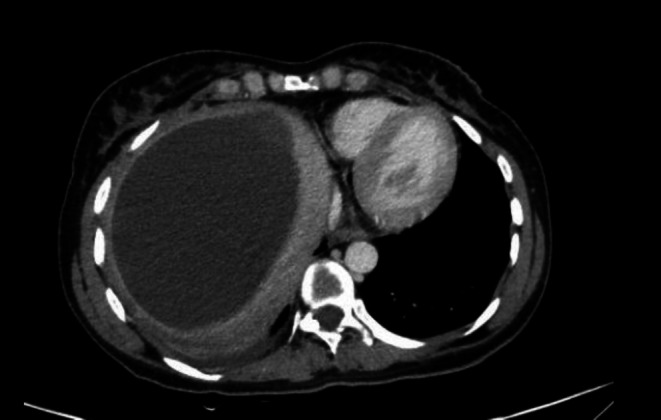
Enlargement of the hematoma after 15 days of discharge.

**FIGURE 5:**
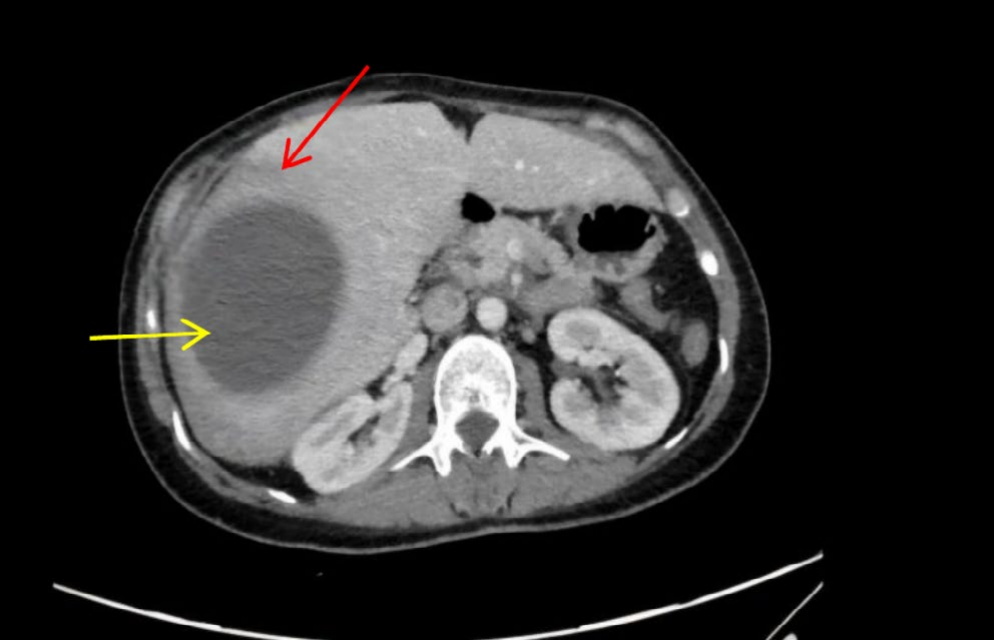
CT scan after the operation and the evacuation of the hematoma.

Fifteen days after being discharged, the patient returned to the emergency department, complaining of RUQ discomfort that had persisted for the previous 5 days. White blood cells were 12.5 × 1.000/μL and hemoglobin was 9.1 g/dL, according to laboratory testing. When a CT scan was obtained, it revealed that the hematoma, which measured 16 × 11 × 15 cm, had enlarged (Figure 4). Prior to surgery, the patient was treated conservatively using the same prior strategy with Cefazolin. Three days after her hospitalization, a laparoscopic cholecystectomy and evacuation of the infected hematoma were planned for her. A drain was installed within the hollow, and the procedure was completed without any perioperative difficulties (Figure 5). After receiving treatment with Meropenem 1 g three times a day for the next 5 days, the patient stabilized in the days that followed. She was then sent home without any more difficulties after taking Ciprofloxacin 1 g daily for 5 days.

## 3 Discussion

ERCP is a diagnostic and therapeutic technique that is typically safe and minimally invasive. Like any intervention, there is a chance of consequences, the most frequent of which is pancreatitis (1%–7%). Acute cholangitis (1.4%) is another consequence hemorrhage (1%), and duodenal perforation (less than 1%) [5, 6, 7]. Rarely, ERCP can result in hepatic abscesses, paralytic ileus, pneumothorax, or pneumomediastinum [8], or SHH [9, 10, 11].

This case report highlights a complex clinical course following ERCP and sphincterotomy in a 32‐year‐old woman with a history of biliary pancreatitis and choledocholithiasis. The patient's presentation, subsequent complications, and management provide valuable insights into the risks and challenges associated with ERCP.

SHH is a rare complication with few cases documented in the literature [11]. This complication is likely underreported, as many patients remain asymptomatic and routine post‐ERCP monitoring is not routinely performed [1]. However, SHH exhibits a broad spectrum of clinical manifestations, including abdominal pain, shoulder pain, anemia, fever, and hypotension. The literature review revealed that abdominal pain is the most frequently reported symptom (82.0%), followed by anemia (55.7%), hypotension (27.9%), fever (18.0%), and shoulder pain (13.1%) [2, 12]. They typically result from trauma to the bile ducts or hepatic parenchyma during procedures such as sphincterotomy or stone extraction. The presence of air within the hematoma, as seen in this case, suggests infection, which likely contributed to the patient's fever and clinical deterioration.

Although the precise cause of SHH is still unknown, two major theories have been put forward. According to the first, when residual stones are removed, the biliary duct extractor balloon applies traction force, which causes liver damage. This force may cause the biliary branches and arteries to burst, which would cause bleeding [9, 13]. The second, most commonly described hypothesis, implicates perforation of the common bile duct by the guidewire used for cannulation [14, 15, 16, 17, 18]. This perforation may damage the adjacent hepatic parenchyma, causing small intrahepatic vessel rupture. Blood infiltration through the hepatic parenchyma, following a centrifugal pattern constrained by the liver capsule, explains the presence of air within the hematoma and its subsequent pathophysiology. The frequent occurrence of infection in such cases may be attributed to the use of non‐sterile guidewires [2].

While most small hematomas are often resolved with conservative management [12]. Large hematomas need greater monitoring and perhaps surgical intervention since they may result in serious mass effect, biliary compression, and infection.

The conservative management strategy that has been applied in this case included intravenous fluids, blood transfusions, antibiotics, fresh frozen plasma, serial monitoring of hemoglobin levels, repeated evaluation of liver function, regular physical examinations, bed rest, and close observation in an intensive care unit [14, 19, 20]. In addition to antibiotic prophylactic therapy, which is always recommended due to the high risk of infection [10, 14]. Conservative management is commonly employed in the early stages of hematoma formation, especially when the hematoma is stable and not causing significant clinical deterioration. Serial imaging was crucial to monitor for changes in the hematoma's size [12], with the absence of significant changes in ultrasound images in the first few days suggesting a relatively stable situation. The development of fever and worsening symptoms after several days raised concerns for secondary infection of the hematoma. Antibiotics played a key role in managing the infection, but surgical drainage of the hematoma was ultimately necessary to prevent further septic complications [2, 21, 22]. Persistent right upper quadrant pain, along with the enlarging hematoma in the CT imaging 15 days after the procedure, suggested a worsening condition. Laparoscopic evacuation of the hematoma and drainage, combined with cholecystectomy, was done. This procedure not only addressed the infected hematoma but also prevented further complications such as biliary fistula or abscess formation. Post‐surgical recovery was uneventful, and the patient was successfully discharged without further issues.

The significance of early detection and treatment of post‐ERCP problems is shown by this instance. Fever and air locules indicated an infected hematoma, which needed to be surgically drained right away to avoid septic consequences. For stable patients with SHH, conservative care is still the primary choice of treatment; nevertheless, prompt surgical intervention is essential when hematomas are chronic, growing, or exacerbated by infection. In this instance, the diagnosis of hematoma growth and infection may have been made more quickly with closer surveillance and serial imaging. Furthermore, the patient's clinical course may have been reduced with an earlier cholecystectomy. When conservative therapies are unsuccessful or there is evidence of hematoma progression, future instances may benefit from earlier surgical intervention.

## 4 Conclusion

This example emphasizes the need for tailored treatment for patients having biliary procedures as well as the necessity of meticulous post‐procedural monitoring after ERCP. For the detection of problems like SHH, early imaging and careful clinical monitoring are essential. Even while conservative treatment is often suitable, surgical intervention is necessary in instances that are difficult or chronic. To further understand the underlying processes and risk factors of post‐ERCP SHH and to provide uniform recommendations for its care, more research is required.

## Author Contributions

**Anwar Zahran:** supervision, writing – original draft. **Amin Khader:** resources, writing – review and editing. **Firas Besharieh:** resources, validation, writing – review and editing. **Saja Amer:** resources, writing – review and editing. **Ahmed Nofal:** resources, supervision, writing – review and editing. **Mohammed AbuBaha:** project administration, writing – review and editing.

## Funding

The authors have nothing to report.

## Disclosure

All procedures performed in this report involving human participants were in accordance with the ethical standards of the institutional, national research committee, and with the 1964 Helsinki Declaration and its later amendments or comparable ethical standards.

## Ethics Statement

The authors obtained verbal and written informed consent from the patient regarding this case and any accompanying images. A copy of the written consent is available for review by the Editor‐in‐Chief of this journal on request.

## Consent

The institution to which this case was admitted does not require approval for writing this case report.

## Conflicts of Interest

The authors declare no conflicts of interest.

## Data Availability

Data will be provided on request from the editor‐in‐chief due to limitations.
